# Natural hydrogels R&D process: technical and regulatory aspects for industrial implementation

**DOI:** 10.1007/s10856-020-06401-w

**Published:** 2020-07-21

**Authors:** Marta Calvo Catoira, Javier González-Payo, Luca Fusaro, Martina Ramella, Francesca Boccafoschi

**Affiliations:** 1 Center for Translational Research on Autoimmune & Allergic Diseases–CAAD, 28100 Novara, Italy; 2 Tissuegraft srl, 28100 Novara, Italy; 3 Telecomunicación, Department of Signal Theory and Communications, University of Vigo, 36310 Vigo, Spain; 4 Department of Health Sciences, University of Piemonte Orientale, 28100 Novara, Italy

## Abstract

Since hydrogel therapies have been introduced into clinic treatment procedures, the biomedical industry has to face the technology transfer and the scale-up of the processes. This will be key in the roadmap of the new technology implementation. Transfer technology and scale-up are already known for some applications but other applications, such as 3D printing, are still challenging. Decellularized tissues offer a lot of advantages when compared to other natural gels, for example they display enhanced biological properties, due to their ability to preserve natural molecules. For this reason, even though their use as a source for bioinks represents a challenge for the scale-up process, it is very important to consider the advantages that originate with overcoming this challenge. Therefore, many aspects that influence the scaling of the industrial process should be considered, like the addition of drugs or cells to the hydrogel, also, the gelling process is important to determine the chemical and physical parameters that must be controlled in order to guarantee a successful process. Legal aspects are also crucial when carrying out the scale-up of the process since they determine the industrial implementation success from the regulatory point of view. In this context, the new law Regulation (EU) 2017/745 on biomedical devices will be considered. This review summarizes the different aspects, including the legal ones, that should be considered when scaling up hydrogels of natural origin, in order to balance these different aspects and to optimize the costs in terms of raw materials and engine.

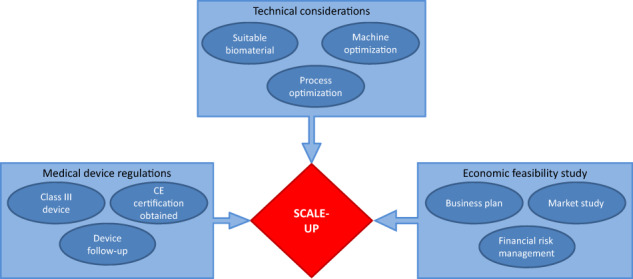

## Introduction

The scale-up process is part of a more complex process of technology transfer from the laboratory to an industrial scale. Scale-up enters into the science of chemical engineering and it attempts the obtainment of a product in the laboratory with the same characteristics and performance on a larger scale. Also, other processing factors, such as the reproducibility of the process or its control, need to be considered. From the normative point of view, in the specific case of the biomedical field, issues such as sterility and the laws that affect these products are important to study throughout the production cycle, from raw materials to the production environment and the chain of material transport. The whole process must be optimized, not only from an engineering point of view but also from an economic point of view, which will be reflected in the economic feasibility study.

This article analyses how the different products obtained from hydrogels of natural origin can be implemented within the biomedical industry, considering the nature of the product and the technology used to obtain the definitive product. Considering the nature of the different hydrogels and the use of different technologies for their final application, the scale-up process varies to suit each need, not only because the technology used for its application should be under consideration, but also because the hydrogel’s nature and its characteristics can define some aspects directly related to the new therapies for its clinical uses.

In order to guarantee the success of the technology transfer process, it is important to foresee the possible critical points and try to minimize the risk that they have on the process and their possible effects.

**Fig. 1:**
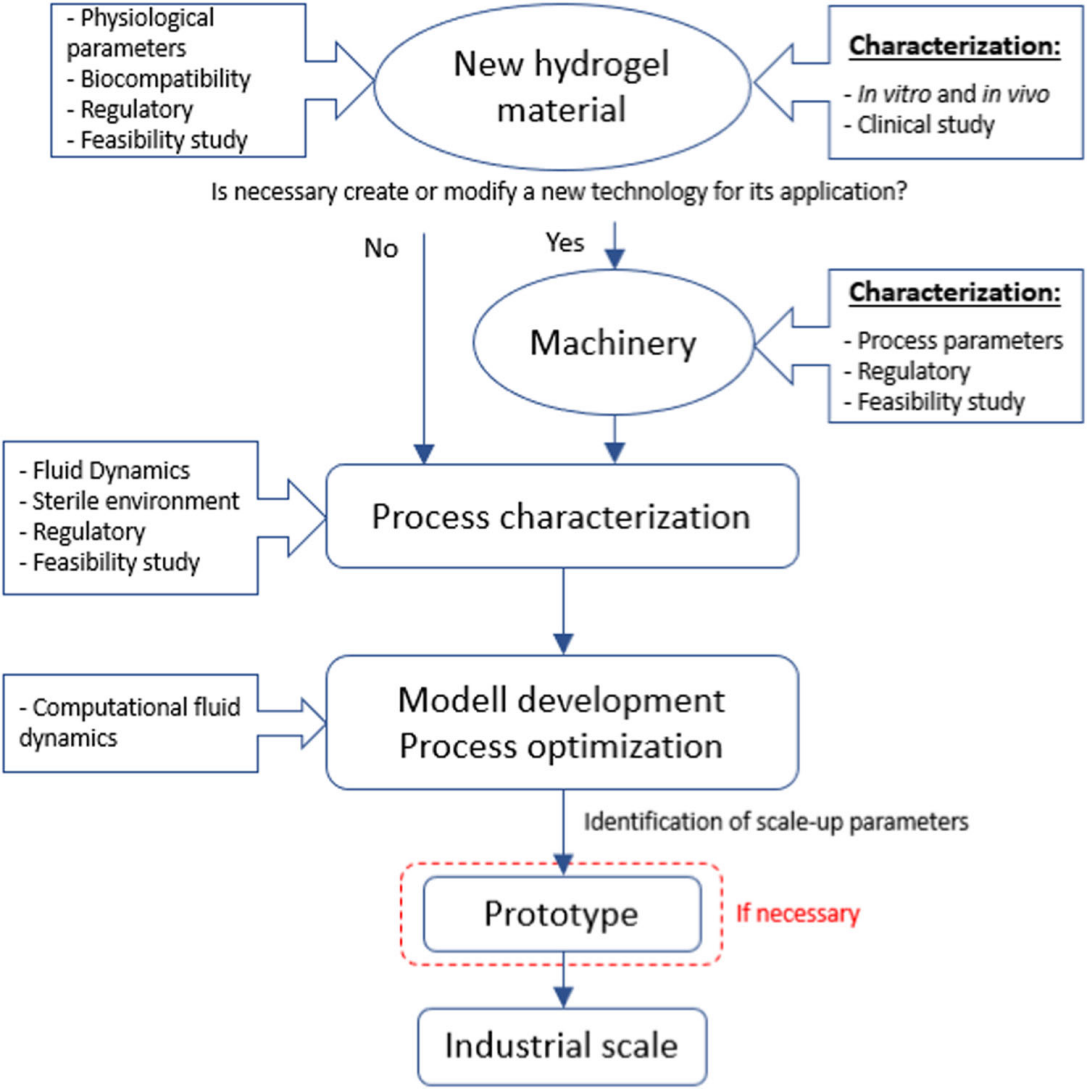
Diagram of the process from the hydrogel obtention in the lab to its industrial production

Figure 1 summarize the relevant phases of the industrialization process and the key points to consider for each step.

## Natural hydrogel characteristics

**Fig. 2:**
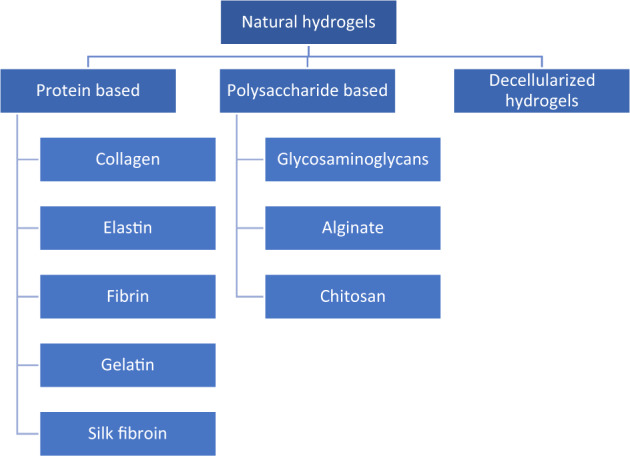
Natural hydrogels materials classification [102]

Based on their composition, natural hydrogels can be classified as hydrogels based on polysaccharides and based on polypeptides (proteins), as a Fig. 2 shows. Each natural material has its own synthesis method, for example collagen is obtained mainly from animal sources in particular bovine, porcine and poultry [1], among them, collagen from bovine source represent around the 35% of total production due to its lower cost production. New source materials are being explored at the moment because of the possible transfer of diseases, such as bovine spongiform encephalopathy (BSE) to humans, discourages its use as a biomaterial in thebiomedical field. Marine-based sources are an alternative but, industrial production cost must be optimized [2–5].

Hyaluronic acid is a glycosaminoglycan and its production is easy an controllable in a large scale due to a microbiological fermentation process, although production costs are higher [6].

On the other hand, fibrinogen can be obtained from the patient’s blood, it allows to create an autologous source for the scaffold obtained in situ in the clinic [7].

The goal of tissue or organ decellularization is to mimic the native tissue and provide a proper environment for cells, through the removal of cells, while preserving the micro-and-macro architecture of extra cellular matrix (ECM) and the niche for cells. When using decellularized tissue as a raw material of hydrogel preparation, the preservation of ECM architecture is not crucial. Polypeptide hydrogels derived from decellularized tissues are the most challenging in the scale-up process due to their recent discovery and their non-implement industrial production at the moment [8]. In particular, some problems presented by the decellularized tissues are: the heterogenity of raw materials, the variability of the digestion process and their temperature sensibility. Decellularized extracellular matrix concentration modifies the material’s flexibility to achieve either paste or putty behavior for in situ placement. It is possible to differentiate a paste and a putty considering their yield stress which directly affects the injectability flow [9].

Natural hydrogels result extremely performant on biocompatibility properties related to cell proliferation and differentiation, however they usually present poor mechanical properties thus they are often combined with synthetic materials or crosslinked with molecules in order to increase their stability [10]. From a rheological point of view, physical hydrogels exhibit moderate yield stress, shear-thinning behavior, and quick recovery time [11]. When combined with synthetic polymers, it is possible to control the degradation rate of the final material if the chemical ratio is balanced [12].

## Hydrogel synthesis: polymerization techniques, crosslink and grafting

As the gelation phenomenon is an important parameter in the reactor design, this must occur under controlled conditions and the process parameters will depend on the final hydrogel use.

Gelation process is usually defined as the transition of a liquid solution into a semi-solid state. In general, the process involves the formation of hydrogen bonds and/or Van de Walls interactions but to increase the mechanical properties or to ensure the stabilization of the gel stronger chemical interactions can be involved [13]. Gelation can occur through two different mechanisms: chemical or physical as indicated in Table 1.

**Table 1:** Classification of gelation mechanism in natural hydrogels and relevant examples [13, 102]

Gelation method	Hydrogel origin	References
Physical methods		
Temperature increase	Chitosan	[103–105]
	Collagen	[103–105]
Cryogelation	Silk	[106]
Chemical methods		
Crosslinkers	Chondoritin	[107]
	Hyaluronic acid	[108]
	Gelatin	[106]
Cation adding	Alginate	[109]

Among chemical mechanisms, crosslinks molecules are the most common. They create covalent bonds between polymers when the two solutions are combined and hydrogel formation follows [14, 15]. The mixing step must be fast and efficient in order to obtain a homogeneous solution and guarantee an appropriate gel formation. Hence, it is important to consider this issue in the reactor design or an external mixing system such as a dual-syringe and mixing tip may be required. Another option is inducing the crosslink reaction using a photo-initiation process [16, 17]. In this case, a UV light is required at the surgical site. If the 3D printing technology is used, an extra laparoscopic hold can be used for the light application.

Radiation-induced gelation presents several advantages like simultaneous gelation and sterilization in a single step of the process, and the easy control of the gamma radiation dose which allows optimal control of hydrogel physical properties [18]. It is also established that the use of this technique, in wound dressings made with a semi-synthetic material, guarantees the stability of quality specifications of the final product up to 24 months. Hence, its fabrication presents a process which is pure and non-contaminated with ballast materials or residuals of toxic initiators.

On the other hand, physical gelation of hydrogels is based on charge, colloidal flocculation, peptide interactions, and physical thermogelation [19–23]. Crosslinks gelation methods have advantages in comparison to the physical gelation methods, such as the process control, but chemical based processes have other advantages such as non-altering the polymer molecules and, in some cases, the process can be reversed [24].

Gelation phenomenon of hydrogels based on polysaccharides and proteins from animal origin occurs when the temperature rises [25]. Temperature modifies the polymer solubility and the formation of packed polymer backbones occurs [26, 27]. Lower critical solution temperature (LCST) and upper critical solution temperature (UCST) are the transition temperatures defined in the case of temperature increase or decrease, respectively, that can produce a thermo-gelation [28, 29]. Gelation time at physiological conditions is a very important parameter for the clinic technology implementation.

## Scale-up key parameters

Usually, literature reports that an hydrogel is injectable based only on the ability to pass through a syringe needle, whereas a precursor solution characterization is important, not only for scaling-up the process but also to correctly inform the clinicians about the real behavior of the gel [30].

Practically, the three most relevant rheological parameters are: (I) easiness of injection (shear response); (II) time for placement (recovery time); (III) retention of the hydrogel precursor solution at the defect site (yield stress) [20].

If it is a thermo-dependent gelation hydrogel, viscoelastic properties should be mapped at varying temperature, and the time required for gelation at body temperature should be characterized. The application of artificial intelligence allows predicting a rheological behavior with time-consuming advantages [31].

Different biological hydrogels may or may not behave like Newtonian fluids. The determination of the fluid’s behavior is important when designing the production equipment since it is one of the variables that will mostly influence the dimensionless numbers that are normally used to scale the production process. In this way, there are functions that standardize rheological properties for both Newtonian and non-fluids. Thus, non-Newtonian hydrogels represent a challenge to predict their behavior.

From the scale-up point of view, there are two reasons to carefully standardize the rheological fluid behavior. The first one is the possibility to predict the flow behavior by comparison with the common standardization function (master curve) which is used as a substance model. The second reason is predicting non-Newtonian fluid parameters based on Newtonian models. Although in vivo performance is always the ultimate metric for translation [32].

In this context, shear stress is defined by dynamic viscosity and shear rate multiplication. In Newtonians fluids, dynamic viscosity depends only on the material and the temperature, but in non-Newtonian fluids, it depends on the effective share rate and, sometimes, on time.

Viscoelastic hydrogels can present dependence of the deformation with the time. These materials can change their shape when an external force is applied and recover the initial conformation after a while. In some kinds of tissues such as vascular, spinal disc and cornea, this parameter is particularly important because they are subject to mechanical loads in physiological conditions [33, 34].

On the other hand, the hydrogel community often uses Herschel-Bulkley equation as a model that allows report hydrogel rheological properties for comparison. This equation simplifies the complex rheological parameters and their interactions [32]. Hydrogel clinical implementation needs a rheological study which is crucial and often overlooked, especially in 3D printing design.

All these characterizations will allow, not only the determination of material injectability, but also the necessary parameters to use the dimensionless numbers needed for the scaling of the production reactor.

## Chemical stabilization

In situ forming natural hydrogels can encapsulate drugs or different substances that guide a functional tissue regeneration, for instance hydroxyapatite and demineralized matrix are used in bone regeneration [9, 35].

Following the global medical tendency, personalized treatments, using hydrogels enriched with patient’s autologous cells, present challenges and limitations. First of all, cell hydrogel enrichment needs a great number of cells that usually exceed the capability of the traditional cell culture methods and expanding setting in a short period of time. Moreover, the phenomenon of homogenization of the gel with the cells is a key parameter for its final application. Although the first steps are explored with good results, this technique must be optimized before its clinical use [36].

Another critical issue is the interspace available in the matrix when crosslink molecules are used to stabilize the gel. As a result, lower cell-cell interactions decrease the migration and cell proliferation, which limits normal cell function and results in less ECM repopulation. Crosslinkers substitution such as formaldehyde, glutaraldehyde, hexamethylene diisocyanate or squaric acid, for example with collagen or elastin, may also be integrated into the hydrogel to supplement for mechanical strength as well as providing a more optimal condition for cell growth and function [37, 38]. Synthetic polymer-free ink used in bioprinting is one of the most interesting techniques in tissue printing, because it allows rapid tissue manufacturing and lacks all the disadvantages associated with synthetic polymers such as degradability, limited cell infiltration and inadequate vascularization. However, mechanical rigidity is the main drawback, so this property must be monitored after bioprinting. In general, the culture of tissue strands for a longer period generates better mechanical properties, but their adhesion capabilities decrease during maturation, therefore, the cell needs to be biologically guided to deposit collagen and elastin in less time. A better mechanical coherency is useful because it helps the operator to load the bio-ink easily, a task that can be further improved with an adequate nozzle design that preserves the mechanical integrity of the threads [19].

## Aspects of designing batch polymerization reactor

Due to their influence on the final product’s characteristics, mixing parameters are important in hydrogels production process. Examining the process production requirements, batch reactor is the best option. However, the classical configuration must be modified considering the necessary aspects in the scale-up process. Fixed time reaction is the main reason for choosing the batch reactor, but high viscosity and small-scale production, due to the high priced products and the high-quality standards, can also justify its use [8].

It is important to remind that most natural hydrogels are obtained in a short time simply by mixing a solid powder phase with different solutions. Mixing must guarantee a homogeneous liquid phase into the batch reactor. With this purpose, Le Cardinal et al. studied three different propellers configurations for a small batch reactor scale to mix the solution depending on the solution viscosity: ribbon mixer with a screw around the axis, screw mixer with four baffles, and double ribbon mixer. These three options obtained good mixing results for Newtonians fluids with high viscosity [39]. To fix the mixing time, Reynolds and Archimedes dimensionless numbers are used. It is necessary to consider the stirrer geometry and speed, and the fluid parameters such as density and dynamic viscosity as well [30].

Regarding the temperature, it should be constant during all the process attending the chemical nature. For instance, in decellularized matrix acid digestion, the temperature is fixed by enzyme activity range and the solution volume can change. In thermal gelation dependent hydrogels, temperature must be lower during the batch production step to avoid the premature gel formation in the reactor. During the reaction, viscosity can change, and it is thermo-dependent. So, this presents a challenge for the process control. Attending the viscosity, power input of the stirrer motor must change to obtain a constant speed of agitation during the process. For that purpose, advanced sensors and suitable software were developed specifically for polymerization reactors [40].

The control of the above mentioned parameters should especially be considered in the case of hydrogels obtained from decellularized materials. The most important reasons are: the production process involves long enzymatic digestion times [41] and during this process the viscosity of the fluid changes. In this context, the reactor mixing device must adapt its power while keeping the temperature below 37 degrees to avoid gelation. In order to guarantee that the temperature of the reactor is lower than the gelation temperature during the whole process, it is necessary to consider the fluid’s viscosity and the consequent heat transport phenomena.

To stop the digestion phase, the solution’s pH will be increased (from 2 to 7), considering the high viscosity of the mixture, the mixing phase must be optimized at this stage.

## Applied technologies

The common method of hydrogel’s application consists in directly applying it on the affected area, spreading on the surface is an example of application in case of burn injury or ulcer treatment or using a syringe that allows its introduction into the body. Among the new technologies which use hydrogels in tissue engineering, electrospinning and 3D printing represent a promising approach although, their industrial implementation still remains a challenge [42, 43].

### Electrospinning

Electrospinning technology consists in the use of the hydrogel as a substrate in the spinning process, in which a certain amount of material leaves a needle with a defined speed and is attracted to an electrode by a difference in electrical potential. The electrode can have different geometries, being flat and cylindrical the most common. Fiber diameter can be controlled through the polymer solution concentration or flow rate. The mentioned electrodes can remain static or rotate at variable speed, which will imply a degree of alignment (fiber alignment) of the fibers of the determined material. Thus, final geometry is also affected [44].

Natural hydrogels of collagen, gelatin, chitin or chitosan used alone or combined with different synthetic materials, such as polycaprolactone (PCL), poly-acrylamide (PAm) and poly-L-lactic-co-glycolide (PLGA) are used to obtain an electrospinning scaffold [43, 45–47]. New hydrogels derived from different decellularized tissues are been employed as a material to create a skin scaffold for regeneration. Kin and co-workers studied the wound healing process using an electrospinning scaffold based on decellularized cardiac tissue and they demonstrated the material’s ability not only to reduce scarring by rapidly replacing collagen type III with the stronger collagen type I, but also the material angiogenesis promotion with a minimum inflammatory response [48]. Other decellularized tissues are also used to create an electrospinning scaffold [49], for instance, muscle tissue [50] but, all of them take advantage of the decellularized material’s ability to induce cell attachment, proliferation, migration, differentiation, and maturation, even of stem cells [51]. The reason is the preservation of natural molecules present into the native tissue, such as growth factors, proteoglycans, bioactive cryptic peptides and natural proteins like collagen and fibronectin, which promote biological activities as cell growth, function, differentiation, angiogenesis, antimicrobial effects, and chemotactic effects [52–54]. Big efforts have been made to control the nanofibres formation through electrically-driven jets into industrial scale [55–61]. Also, a suitable research about complex interconnections between processing conditions/parameters and final products will lead to technical solutions for optimizing costs reliable transfer of electrospinning plants. Thus, electrospinning technology is currently really close to industrial implementation [62].

The entire electrospinning process takes place within a chamber where the environmental conditions, mainly humidity and temperature, are precisely controlled because they directly affect the performance of the material. This fact facilitates the technology scaling process because when the production device is positioned inside the white chamber, the environment is confined. The control into the cleanroom of the possible particles that can be detached from the process is facilitated by the confinement within the electrospinning chamber. In addition, the problems of bioink sterility are reduced given the voltage to which the material is subjected.

The manufacture of the material to be electrospinned using natural materials usually takes between 2 and 4 h [63]. But in the case of decellularized materials, the obtainment of the solution to be electrospinned can last much more time, about 24 h depending on the tissue.

### Bio-printing

Electrospinning is a conventional production of scaffolds with good results. However, it is difficult to obtain pre-determined, well-defined architectures in a controlled manner.

The 3D printing technology that uses biomaterials, specifically natural hydrogels such as bioinks, presents more complex problems when it comes to scaling the process. Hydrogels that are sensitive to temperature changes make the in-situ production of the material more likely, profitable and safe.

Natural hydrogels are promising bio-inks for 3D printing scaffolds for regenerative medicine in different materials like gelatin, chitosan or alginate, that promote tissue regeneration such as bone or cartilage [64–69].

Bioinks should present desirable characteristics such as, biocompatibility (not cause any immune or undesirable response after implantation), printability (as printing materials), and good mechanical properties (resist physical forces of the environment) [70].

Due to the big effort to fix the cause of failure of traditional scaffolding methods, which concerns the efficient media transportation into the scaffold, new 3D printing technologies increased progressively their complexity from the engineering point of view [71]. In this context, the integration of the vascular system into the scaffold structure is the best option. This embedded microfluidic network allows oxygen, growth factors, and water transportation, but its fabrication needs a hybrid biomimetic design fabrication approach, which involves advanced print techniques [19].

Beyond the benefit of paste and putty hydrogel precursor solutions for surgical use is the benefit of their direct applications in hydrogel bioinks for 3D printing [11, 20, 72–74].

Hydrogel precursor can easily flow through a needle gauge depending on its diameter. Here, it is possible to difference its final application: an injectable material (flowing through a needle, smaller diameter than a syringe) could have applications in laparoscopic surgery, whereas syringeable material may need open surgery for placement [32].

The flow of a material depends on the shear response obtained after applying a force in function of the flow rate. During the application of that force, the material can suffer shear thinning, if it decreases the apparent viscosity when the stress is increased, or a shear thickening, if the apparent viscosity is increased when the stress is decreased. The apparent viscosity can change with stress as explained, and also with time, being thixotropic when it decreases with time, or rheopectic if it increases. For injectable biomaterials, the property of thixotropy is more convenient in case that the process lasts less time than needed for injection. Hydrogel precursor solutions normally have a shear thinning behavior, which facilitates the delivery from a syringe due to the shear rate increase in the needle, shear-thinning reduces the apparent viscosity and, therefore, the resistance to flow.

For the sake of reducing the gap between rheology and clinical relevance, the connection between injectability/syringeability and shear response, material placement and recovery time, and material retention and yield stress are helpful. A summary of rheological properties published allows to compare hydrogels [32].

Bioinks for 3D-printing applications that exhibit shear thinning behavior, fast recovery time (i.e., >85% of G’ within 5–10 s), and presence of a yield stress (>100 Pa) are adequate for 3D bioprinting applications.

Three-dimensional printing technology avoids the limitations imposed by conventional scaffold techniques, allowing the production of patient-specific scaffolds. Four-dimensional printing is an innovative technique in tissue engineering that, using smart materials, fabricates scaffolds that mimic the dynamic nature of tissues to a very large extent.

A new category of printing has recently been introduced in the biomedical field, called 4D printing. Initially, it started as a new technology in the construction and manufacturing field and it was defined by the ability for a material to change form and/or function after printing [75].

Hydrogels from decellularized matrix are dynamic biomimetic scaffolds that transmit biochemical and mechanical signals from the microenvironment into the cells and affect cell behavior. For this reason, it is considered a smart material used to make the 4D scaffolds [75–77]. Introduction of the fourth dimension, time, in addition to the 3D arrangement gives both spatial and temporal control over the fabricated product.

The environmental control process defines the production plant location. This step could be considered from the beginning of the hydrogel characteristics, their final use and the technology used for the application.

The question of whether to produce the gel with a mobile reactor adapted to the bioprinter in situ is controversial but the best option will always be to ensure that the hydrogel produced maintains the optimum characteristics for its performance.

The preservation of the hydrogel during the transport phase, from the production plant to the hospital, can be a challenge depending on the nature of the gel. Hydrogels with an application such as bioinks, in which gelation phenomenon occurs considering the temperature, are usually very sensitive to small variations in the operating parameters in the production phase. Therefore, some materials, such as hydrogels derived from decellularized tissues, are capable of being manufactured in situ in a reactor adapted to a controlled environment but within hospital facilities. Thus, the starting material can be provided by the producer in a kit format that the qualified operator will use to give rise to on-site production.

It is possible that the new technologies will require professional figures that do not currently exist, a bioprinter expert who works in a hospital may be necessary. This person should have knowledge, not only of computer science and robotics, but also of the optimization of the bioink production process depending on which one it is, and the necessary requirements in its production process. For this reason, it is probable that an area in the hospital dedicated to the printing of scaffolds or even an operating room enabled for it should be made available.

The standardization and control process in such environment will also require standard quality control tests that must be standardized by the company that provides/produces the bioprinter and the bioink production or conditioning upstream for its different applications.

## Regulatory

Since hydrogels are considered a medical device class III by the new European regulation, this regulation must be considered not only in the scale-up process but also in the initial phases of the material’s and the machine’s invention. In this section, the most relevant changes in the regulation, which concernclass III devices, will be summarized and compared with the previous regulations. Also, the process to obtain the device commercialization approval will be summarized

*Summary of the laws* The commission regulation (EU) N0 722/2012 of 8 August 2012, concerning particular requirements as regards the requirements laid down in Council Directives 90/385/EEC and 93/42/EEC with respect to active implantable medical devices and medical devices manufactured utilizing tissues of animal origin, has adopted a regulation based on: the original requirements, the maintenance of a high level of safety and health protection against the risk of transmitting animal spongiform encephalopathies.

The regulation also considers that the active implantable medical devices and medical devices of class III are subject to the conformity assessment procedures, prior to being placed on the market or put into service, demanding the adoption of more detailed specifications relating to the risk analysis and management.

It is considered also for the elaboration of the regulation, the convenience of laying down additional provisions on the use of animal by-products not intended for human consumption, the adoption of several opinions on specified risk materials and on minimizing the risk of transmitting animal spongiform encephalopathy agents.

The regulation considers that it is appropriate for the Member States to verify that the notified bodies, designated to assess the conformity of those medical devices, have all the necessary expertise and up-to-date knowledge to perform this task.The period for scrutiny granted to the competent authorities of the Member States in relation to the notified bodies’ summary evaluation report should be shorter for medical devices manufactured using starting certified materials than in cases where uncertified materials are used. The regulation also bases its decisions on the convenience of the provision of an adequate transitional period allowing for active implantable medical devices already covered by an EC design-examination certificate or by an EC type examination certificate to continue to be placed on the market and put into service, and that the measures provided for in this Regulation are in accordance with the opinion of the Committee on Medical Devices.

Based on these considerations, the regulation establishes particular requirements in relation to the placing on the market and/or putting into service of medical devices, including active implantable medical devices, manufactured utilizing animal tissue and their derivatives, originating from bovine, ovine and caprine species, deer, elk, mink and cats. In the case of collagen, gelatin and tallow used for the manufacturing of medical devices they shall meet at least the requirements as fit for human consumption.

The regulation also establishes that the manufacturer of medical devices or his authorized representative shall carry out the risk analysis and risk management scheme before lodging an application for a conformity assessment.

Member States shall verify that bodies have up-to-date knowledge of the medical devices in order to assess the conformity of those devices, and shall take all necessary steps to ensure that medical devices are placed on the market and/or put into service only if they comply with the current provisions and the particular requirements laid down in this Regulation.

Conformity assessment procedures for medical devices shall include the evaluation of compliance of the devices with the essential requirements into the current directives and the particular requirements laid down in this Regulation. The manufacturer shall collect, evaluate and submit to the notified body information concerning changes regarding the animal tissue or derivatives used for the device or regarding the risk in relation to the device.

The new two European Health Products Regulations came into force on May 26th 2017. The first one, the Regulation (EU) 2017/745 of medical devices, which modifies Directive 2001/83/EC and subsequent derived regulations, repealing Council Directives 90/385/EEC and 93/42/EEC, will be applicable from May 26^th^, 2020. The second one, Regulation (EU) 2017/746 of in-vitro medical devices, which repeals both Directive 98/78/EC and the decision of the commission 2010/227/EU, will applicable from May 26, 2022.

The new regulations represent an important change in the medical devices field and will require new and stricter obligations to all market operators, in order to achieve an increase in transparency and traceability guarantees on the market.

This is the detail of some of the most important changes and novelties contained in the Sanitary Products Regulation:

- *Transitional period*: Since a transitional period of three years is expected from its entry into force, since May 26 2017, and until May 26 2020, both regulatory frameworks may coexist and certificates may be issued in accordance with the provisions of the new Regulation or the old Directives 90/385 / EEC and 93/42 / EEC. In the latter case, the certificates will have a maximum validity of 5 years and they will be considered invalid as of May 27, 2024. In any case, any product introduced on the market in accordance with the requirements of the old Directives can only continue to be marketed or be put into service until May 27, 2025.
- *Specification of the definition of health products*: The definition of “health product” is specified and defined. The definition includes clearly computer programs, implants or reagents, and establishes the need that the manufacturer has foreseen a specific medical purpose, admitting new purposes such as “prediction or prognosis of diseases or research, substitution or modification of a physiological or pathological state”. In addition, the Regulation clarifies that sanitary products, “control products or support for conception”, as well as products specifically intended for cleaning, disinfection or sterilization of medical devices and their accessories are within the scope of this Regulation. The 3D printer and even the software that controls the printing process fall within this regulation.
- *Inclusion of products without medical purpose*: One of the great novelties is the inclusion of series of products without medical purpose that must also comply with the provisions of the Regulation. These are basically esthetic products (contact lenses, substances or articles intended for uses as a facial filler or in other dermal areas, liposuction equipment, lipolysis or lipoplasty, equipment that emits high intensity electromagnetic radiation intended for use in the human body, and equipment destined for brain stimulation) that although they do not have a medical purpose, it has been considered that their characteristics and risk profile is comparable to medical devices and, therefore, they must meet the same requirements. These products are included in Annex XVI of the Regulation and must comply with common specifications that must be approved by the Commission before May 26, 2020.
  In accordance with the Regulation, this type of product must comply cumulatively with the regulation of medical devices and with the regulation of products without medical purpose, which may imply some practical difficulties.
- *Increased transparency and traceability:* New measures are introduced aimed at increasing transparency and traceability:
  1. A European Union Global Database (EUDAMED) for medical devices is created, a European portal that is essential for the practical application of the Regulation since it must include the new electronic systems created by the Regulation: the electronic registration system of products, the Unique Product Identification (UDI) database, the electronic system for registering economic agents, the monitoring and post-marketing monitoring system and the electronic market control system. It will be part of the mandatory Quality Management System (QMS) demanded by the law. This database must be up and running by March 25, 2020. Hydrogels implants cannot allow an ink traceability mark so, a traditional database of the patents must be implemented.
  2. The Product Registry is created and the Unique Product Identification system (UDI) is established to guarantee the traceability of the products at any time, which obliges manufacturers to assign and maintain an UDI for all their products. This item represents a challenge in scaffolddevices, due to the impossibility to include a mark in some materials,such ashydrogels. In hard tissues such as bone prosthesis, a code can be printed during their production but in soft tissues applications like a hernia patch or a vessel scaffold, a suitable ink must be created.
  3. The Registry of economic operators is created in which all manufacturers, authorized representatives and importers must register before introducing a sanitary product on the market.
  4. All manufacturers of implantable products are obliged to accompany the implant card product and information that allows the identification of the product, the warnings or measures that the patient or health professional must take, and the regulation also obligates the Member States for demanding health centers to provide the mentioned card and the required information to patients. It is highly recommendable to include theclinical data sourced from clinically relevant information coming from post-market surveillance, in particular from the post-market clinical follow-up.
- *Reinforcement of guarantees in all stages:* The pre-commercialization and post-marketing guarantees of medical devices are reinforced. In relation to pre-commercialization guarantees, the role of the European Commission of categorizing and classification of products is increased, and it should no longer be limited to action if a Member State previously requests it, but it must do so *ex officio*. For this purpose, the Member State will count on the help of a Coordination Group of Health Products with European experts.
  On the other hand, the threshold of demand for the introduction on the market and the commercialization of products with more rigorous requirements in the procedures for the designation and supervision of the Notified Bodies evaluating the products, and in the conformity assessment procedure, is raised. In the specific case of high-risk products, such as medical implants, it will be necessary to consult with a panel of European experts before being able to introduce them into the market.
  The post-marketing guarantees are: establishment of the electronic surveillance system which requires the notification of serious incidents and corrective actions, obligation of manufacturers to have an adequate post-marketing monitoring system for each product that allows to improve the determination of the benefit-risk ratio, risk management, updating of information on the design, manufacturing, instructions for use, preventive measures and trends detection, and the obligation to issue a report on post-marketing monitoring.
  In addition, with the aim of ensuring greater and better control of the market, greater discretion is granted to the authorities, who may request additional information or review the technical documentation made by the Notified Body for the products class III and class II B, and they will even be allowed to perform a certain number of on-site audits.
- *Definition of obligations of the economic operators:* The obligations and responsibilities of each one of the economic agents that were not sufficiently detailed in the previous regulation, are here specified in detail; forcing manufacturers to establish a quality assurance system, a system to obtain and review data after commercialization of products, to establish measures that offer sufficient financial coverage adapted to the type of risk, type of product and size of the company, as well as having a technician responsible for compliance with the regulations, who may be trained in Law, Medicine, Pharmacy, Engineering or other scientific discipline, and one year of professional experience in regulatory matters or in quality management systems related to medical devices.
- *Clinical research:* Another change that will have a strong impact on manufacturers concerns the process of developing new healthcare products, which must comply with new and more stringent requirements. Following the policy of transparency desired by Europe, manufacturers should make public the results of the clinical evaluation in the case of implantable and class III products. Likewise, the use of clinical evaluations is restricted by equivalence and, in the permitted situations, the conditions for it are hardened. In addition, the authorization and notification process for multicenter trials is unified and centralized and a single Member State is allowed to serve as the representative of all the Member States involved in the trial and as an interlocutor with the rest of the members. The rights of people participating in a clinical trial are reinforced.
- *Necessary supplementation and national regulation:* The Regulation that entered into force requires subsequent actions so that its application can be complete. Thus, the text itself foresees the need for delegated acts and regulations to be adopted by the Commission in a second phase. Specifically, up to 80 regulations will be necessary, 18 of which are considered indispensable, and essential for the application of the Regulation.

But, in addition, and despite the fact that the Regulation establishes a specific legal framework and to be directly applicable in all Member States, it is necessary for the national authorities of the Member States to regulate several issues that the Regulation itself entrusts to them, such as the linguistic aspects of the labeling of products, the permissibility and regulation of single-use products and their reprocessing, the conditions for the use of in-house products, the compensation mechanisms for damages in case of defective products, as well as the distribution of competencies between the state agency and the autonomous communities.

Ultimately, after the Regulations come into force, a new stage is opened in which it will be necessary to be attentive to all the acts of application that are dictated both at national and European level.

*Table of main differences between the former and the new law*

**Table:** 

*Changes*	*Former law until May 26th, 2017* *(EU) No 722/2012*	*Law from May 26th, 2020* *(EU) 2017/745 – 2017/746*
*Authorities: Notified bodies*	*“Industry partners” roles*	“Policing bodies” roles: - Unannounced audits - Maintaining their notification - Special notified bodies: for high-risk medical devices
		*Implementation of expert committees*
*Manufacturers*	Definition: - Handles only manufactured products	Definition: - Handles also fully refurbished products
	Responsibilities: - Maintain a Technical File - Quality management system	Characteristics: - Qualified Person - Specific Annex II for technical documentation - Process of clinical evaluation - Market surveillance - Involvement of authorities and/or expert groups in the market approval - Common standards
		Responsibilities: - Maintain a Technical File - Quality management system (including continuous product improvement)
		Obligations: - Technical file - Declaration of conformity - UDI system; Registration - Labeling
*Technical file maintenance*		New points: - Filed since the last device was placed on the market for at least 10 years (all devices), 15 years (implantables) - If the manufacturer is outside the EU, this is a responsibility of the EU REP
*Transparency*		*Creation of European Union Global Database (EUDAMED)*
*Devices with a non-medical purpose*	*Not included*	- “Cosmetic” contact lenses and other devices intended to be inserted in the eye - Cosmetic surgery fillers (excluding tattoo ink) - Equipment for liposuction - Light emitting equipment for cosmetic use (tattoo or hair removal) - Brain stimulators - List may be integrated over time (Article 1.c)
*New definition of medical device*	*Includes medical devices of control of conception*	- Eliminates medical devices of control of conception - Includes medical devices for providing information by means of in vitro examination of specimens derived from the human body, including organ, blood and tissue donations
*Falsified device*	*Not included*	False presentation of identity, source, … - False documentation for CE mark - Does not include un-intentional NCs
*User related definitions*	*Not included*	User: healthcare professional or lay person that uses the device - Lay person: person with no formal education in the field of use of the device
*Quality management system (QMS)*		*Improved contents: Plans, procedures, surveillance, communication and improvements*
*Classification rules*	*18 rules in total*	- 23 rules in total - Risk assessment of the intended time of use
*Classification path*	- Invasiveness - Special rules - Additional rules	- Device identification - Length of use - Invasiveness - Source of energy - Special rules

*European Union medical device approval process*

*Every medical device must comply the European regulation for that type of product, and to obtain the CE mark it must be in line with the requirements established for that type of device. The process adapted for hydrogels is as follows:*

1. *Determination of the regulation that applies, taking into account that hydrogels are active implantable medical devices*.
2. *Classify the medical device: Class I, IIa, IIb or III. Hydrogels are Class III*.
3. *Implementation of the Quality Management System*.
4. *Elaboration of a technical file with all of the available information, including physical, chemical and technical characteristics and properties, and clinical data about the hydrogel in order to prove its compliance with the regulation*.
5. *Identification of an Authorized Representative located in Europe fully qualified in European regulatory issues in case that the manufacturer is not based in Europe*.
6. *Audit from a Notified Body of the QMS and technical file of the hydrogel. The clinical evaluation reports and the post-marketing surveillance activities must be also performed*.
7. *Obtention of the CE marking certificate (valid for three years) for the hydrogel and an ISO 13485 certificate (valid for one year) for the manufacturer facilities, if the former audit is successful*.
8. *Elaboration of a Declaration of conformity declaring the compliance of the hydrogel to the corresponding regulation, where the CE marking certificate can be now attached*.
9. *Registration of hydrogels in those member states where their national regulation requests it because it is only mandatory for Class I devices*.

*The process will be repeated once the CE mark/ISO 13485 certificate lose their validity*.

**Fig. 3:**
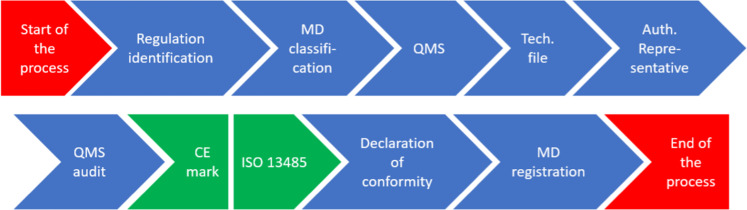
Flow of the European Union medical device approval process

*The* Fig. 3*shows the flow of the described process*.

## Financial remarks

The most common natural material used in the biomedical field is collagen, although the high cost to produce pure collagen in large scale is not cost-effective and consequently curtails its utilization [78]. Alternative commercial natural hydrogels, based on fibrin, chitosan or hyaluronic acid are also expensive [79] thereby, big efforts are being made to obtain an alternative suitable material. In this context,the financial aspects, involved not only in the industrial plant construction or technology cost but also in the operative cost of the production plant, should be considered. If a feasibility study is created from the initial phases of the process the success of the project will be increased.

Economics has always been part of engineering, and many companies, especially those dealing with new, innovative or risky projects, employ advanced financial tools from early steps in their decision making. Those tools are not widely used nevertheless, only certain aspects of the tools that economists and financiers use, namely a few profitability measures, are fully integrated into the engineering environment. This fact has started to change in recent years, but many tools are still completely out of sight for mainstream chemical engineers.

In terms of, for example, the profitability, the engineer is expected to determine the right capacity and the level of flexibility that is appropriate for the design, maximizing the expectation of profit. The profit measures, however, did not change.

Now, the engineer should be able not only to plan the evolutions of profitability, but also to incorporate the uncertainty as a variable to be considered. In any case, projects are completely different from each other because some are constrained by the costs, some by regulations, and the rest of them by other factors. Therefore, the type of economic analysis and its associated tools change.

Engineers need to address some questions related to financial risk, impacts on projects due to shareholder value or cash position, decisions related to the size of the company or how to consider exogenous parameters like prices or demands in the final profitability. The project evaluation for chemical engineers uses the following measures of profitability:

- *Internal rate of return*: The internal rate of return (IRR) is used to estimate the profitability of possible investments. IRR is designed to account for the time preference of money and investments. A given return on investment obtained at a given time (higher IRR) is better than the same amount received at a later time (lower IRR), if all the otherfactors are equal [80].
- *Pay out time*: It is the expected financial return from an investment. It may be expressed on a period or as a percentage of the investment’s cost. Payout can also refer to the period in which an investment or a project is expected to recover its initial capital investment obtaining a minimum profit.
- *Net present value (NPV)*: It provides a method for evaluating and comparing capital projects or financial products with cash flows spread over time, as in loans, investments, payouts from insurance contracts plus many other applications [81].
- *Discounted cash flow rate of return*: To apply this method, all future cash flows are estimated and discounted by using cost of capital to give their present values. The sum of all future cash flows, both incoming and outgoing, is the net present value (NPV), which is taken as the value of the cash flows in question [82].

Although, the project evaluation considered some old conceptions, due to the impossibility to measure the uncertainty with not so powerful computers. Uncertainty in project evaluations has been handled through decision models, trees, payoff tables [83–85], and lately dynamic programming [86].

Dynamic programming is a mathematical optimization method developed by Richard Bellman in the 1950s with many applications in different areas. The goal of the method is the simplifying of a complicated problem by splitting up into simpler problems in a recursive manner. This type of programming is a good technique to solve sequential decision-making processes, and it is equivalent to two-/multi-stage stochastic programming, which is a method for optimization based on uncertainty, with the added benefits that under certain conditions, some properties of the solutions are known and are helpful for the solution procedure. So, chemical engineers have understood uncertainty and flexibility and have incorporated them within a two (multi)-stage process decision optimization model, achieving an integration of financial indicators other than financial risk as well as strategic planning as a whole.

Economists and financiers evaluate projects in a different way, maximizing dividends of the company and using other key issues: risk management and diversification, cash flow management, and liquidity. All these indicators focus on different aspects of the enterprise, but they should be at least used as constraints in engineering models. It is therefore imperative that engineers incorporate these measures and objectives in project evaluation. A major component influencing business decisions is risk. Business risk is measured by the non-dimensional ratio of variability to expected profit before taxes and interest [87, 88], and financial risk is defined as the “additional variability in earnings and the additional chance of insolvency caused by the use of financial leverage” [87]. Engineers have not yet caught up in relating these concepts with their models. Financiers only know how to evaluate a project. They can manipulate it on the financial side, but they cannot manipulate it in its technological details because they need engineering expertise for that. Engineers, in turn, cannot easily take into account the complexity of finances. Both need each other more than ever.

The chemical engineering models have evolved from different techniques such as resource allocation, scheduling [89–91], dynamic programming [91, 92] and risk management, to new emerging procedures like decision trees and utility theory, or multi-objective Markov decision processes [93]. A Markov decision process is a discrete time stochastic control process that provides a mathematical framework for modeling decision making in situations where results are partly random and partly under the control of a decision maker [94].

Additionally, there are some points that should be considered:

- Effect of inventories on financial risk: The effect of inventories can be better appreciated through the analysis of the risk curves [95].
- Effect of option contracts: A future contract is an agreement to buy or sell an asset at a certain time in the future for a certain price. The risk curve analysis determines that option contracts do not automatically reduce risk and require risk management, and they are excellent tools to reduce risk at low profit expectations, maintaining upside potential [96].
- Effect of regulations: The effect of regulations (bilateral and multilateral international trade agreements, import tariffs, corporate taxes in different countries, etc.) impacts on the capacity expansion problem [97].

Traditionally, pricing policies considered in an integrated manner with scheduling decisions increase profit [98]. But this model does not always converge. Moreover, an alternative model in which process and production schedules are obtained simultaneously is proposed. The integrated model of operations planning and pricing produces different schedules and prices and allows larger profits.

There are other factors that are involved into the project evaluation like the consumer satisfaction [99], whose evaluation must take into account the interrelation between three different objectives: NPV, customer satisfaction, and financial risk. Also, it is important to remark the relation between product engineering and process engineering. Process engineering, which is mistakenly associated with the production of commodities, is an integral part of product design. Product design cannot exist without process design. So, there is no antagonism of any sort. Additionally, when the chemical supply chain is considered in the context of process design, one realizes that the chemical supply chain contains many of the elements of product design.

The last aspect to be considered is the environment [100]. Some authors incorporate sustainability and the global life cycle assessment of processes and products in process design, but others have opted to leave sustainability as an alternative objective to be considered together with profitability and perhaps financial risk, but the discussion is still open.

As conclusion the full integration of several disciplines – management, finances, industrial engineering, and chemical engineering, among others – is slowly taking place. Further details about this combined approach are available on Galán MA et. Al, 2010 [101].

The result is a “beginning to end” modeling of the product research/development and delivery supply chain. In turn, more interactions with other disciplines will come. At some point, with powerful computers and adequate modeling, one can dream of the whole process being fully integrated into one single model.
